# Evolution of Plant Architecture, Functional Diversification and Divergent Evolution in the Genus *Atractocarpus* (Rubiaceae) for New Caledonia

**DOI:** 10.3389/fpls.2018.01775

**Published:** 2018-12-04

**Authors:** David Bruy, Tom Hattermann, Laure Barrabé, Arnaud Mouly, Daniel Barthélémy, Sandrine Isnard

**Affiliations:** ^1^AMAP, IRD, CIRAD, CNRS, INRA, Université de Montpellier, Montpellier, France; ^2^AMAP, IRD, Herbier de Nouméa, Nouméa, New Caledonia; ^3^Endemia, Plant Red List Authority, Nouméa, New Caledonia; ^4^Laboratoire Chrono-Environnement UMR 6249 CNRS, Université Bourgogne Franche-Comté, Besançon, France; ^5^Jardin Botanique de la Ville de Besançon et de l'Université de Franche-Comté, Besançon, France; ^6^CIRAD, UMR AMAP, Montpellier, France

**Keywords:** branching index, convergence, corner's rules, gardenieae, Island, rainforest, treelet, understory

## Abstract

The diversification of ecological roles and related adaptations in closely related species within a lineage is one of the most important processes linking plant evolution and ecology. Plant architecture offers a robust framework to study these processes as it can highlight how plant structure influences plant diversification and ecological strategies. We investigated a case of gradual evolution of branching architecture in *Atractocarpus* spp. (Rubiaceae), forming a monophyletic group in New Caledonia that has diversified rapidly, predominantly in rainforest understory habitats. We used a transdisciplinary approach to depict architectural variations and revealed multiple evolutionary transitions from a branched (Stone's architectural model) to a monocaulous habit (Corner's architectural model), which involved the functional reduction of branches into inflorescences. We propose an integrative functional index that assesses branching incidence on functional traits influencing both assimilation and exploration functions. We showed that architectural transitions correlate with ecologically important functional traits. Variation in ecologically important traits among closely relatives, as supported by the architectural analysis, is suggestive of intense competition that favored divergence among locally coexisting species. We propose that Pleistocene climatic fluctuations causing expansion and contraction of rainforest could also have offered ecological opportunities for colonizers in addition to the process of divergent evolution.

## Introduction

Convergent and divergent evolution are widely recognized as important drivers of plant diversification at large scale (Givnish and Sytsma, 1997; Gianoli, 2004; Drummond et al., 2012; Couvreur et al., 2015; Givnish, 2016). Well known examples of large scale convergence in plants (i.e., the appearance of phenotypic similarities among distantly related taxa) include the evolution of a cushion growth habit in alpine environments (Boucher et al., 2012, 2016; Aubert et al., 2014), rosette-shrubs in islands (Carlquist, 1974; Givnish, 2010; Lens et al., 2013), climbing mechanisms in lianas (Sousa-Baena et al., 2014, 2018), and succulence in arid environments (Ogburn and Edwards, 2010; Arakaki et al., 2011). These and others examples demonstrate that similar habitats or micro-habitats can produce selective pressures that favor some morphological and physiological traits. Another evolutionary process, sometimes linked with convergence, is the diversification of ecological roles among closely related species, i.e., divergent evolution, which results in the exploitation of different ecological resources (e.g., Bramwell, 1975; Givnish et al., 2009, 2014). Convergent and divergent evolution have been attributed to morpho-physiological traits, whose gradual evolution or rapid innovation can lead to adaptive radiation, sometimes involving increased diversification rates (Givnish and Sytsma, 1997; Givnish et al., 2014; Couvreur et al., 2015). A striking feature of convergent and divergent evolution is that it often involves variation in growth habit, which represents the ultimate form of a plant expressed in its physiognomy (Warming, 1909). Growth habit results from the integration of a set of traits, e.g., branching pattern and structure, body size and shape, position of inflorescences, and anatomy, among others, which have generally been studied independently (e.g., Carlquist, 1984; Rowe and Speck, 2005; Givnish et al., 2009; Isnard et al., 2012; Wagner et al., 2014). Few work has, however, used the integrative approach provided by plant architecture to study the evolution of plant growth habit.

Plant architecture characterizes the spatial arrangement and specialization of structures (morphological origin, branching pattern, axis categorization) and their evolution during ontogeny (Hallé et al., 1978; Barthélémy and Caraglio, 2007). As such, it can highlight how plant structure impacts plant diversification and can help identify evolutionary processes underlying plant evolution (Bateman, 1994, 1999; Sussex and Kerk, 2001; Meyer-Berthaud et al., 2010). Architectural studies have shown that plants are modular organisms comprising elements that can differ in their organization and functions (Hallé et al., 1978; Barthélémy and Caraglio, 2007). For instance, in many tree and treelet species, the trunk functions primarily for exploration and to provide support, while branches are involved in assimilation and reproduction. Plant architecture thus influences spatial and temporal exploitation of resources (Smith et al., 2014). Some architectural traits have been shown to impact plant fitness, either directly (Küppers, 1989; Millet et al., 1999; Charles-Dominique et al., 2010, 2012, 2017; Millan, 2016) or in interaction with other functional traits (Pérez-Harguindeguy et al., 2013; Trueba et al., 2016). Among the best known examples are Corner's rules, which stipulate that “the greater the ramification, the smaller become the branches and their appendages” (leaves, flowers, and fruits, Corner, 1949). This statement points toward an effect of plant architecture on leaf size and linked architecture and plant ecological strategies (White, 1983a; Ackerly, 1996). To date, approaches linking plant architecture and function are scarce although some architectural indexes can assess interesting strategies such as defense against herbivores (“Index of caginess,” Charles-Dominique et al., 2017) or leaf-to-stem relation that trigger flowering (“Index of axialisation,” Lauri, 1988; Lauri and Kelner, 2001).

Despite the functional importance of plant architectural traits, little is known about their evolution (Kurmann and Hemsley, 1999; León Enriquez et al., 2008). In a recent review, Chomicki et al. (2017) investigated the evolution of plant architecture for several groups. Their results showed preferential transitions between models, suggesting pre-requisite morphological ability for the evolution of a given plant architecture. In contrast, some model transitions seem never to occur, suggesting that genetic constraints might prevent them. Chomicki et al. (2017) did not, however, investigate processes involved in plant architectural evolution. Bateman (Bateman and DiMichele, 1994; Bateman, 1999) argued that the changes involved in the transition from one architecture to another are too important and deep for such transitions to take place gradually. Rather, such evolution is suggested to occur preferentially by saltation, which involves the direct shift from one competitive architecture to another without passing through intermediate states associated with lower fitness. Other authors have proposed the notion of an “architectural continuum” (Oldeman, 1974; Barthélémy et al., 1989), suggesting a continuous transition from one model to another in some groups (Hallé et al., 1978). In the context of this diversity of possible models to explain evolution in plant architecture, detailed studies within individual clades that combine results from molecular phylogenetic work with detailed architectural analysis may be particularly informative.

**Figure 1:**
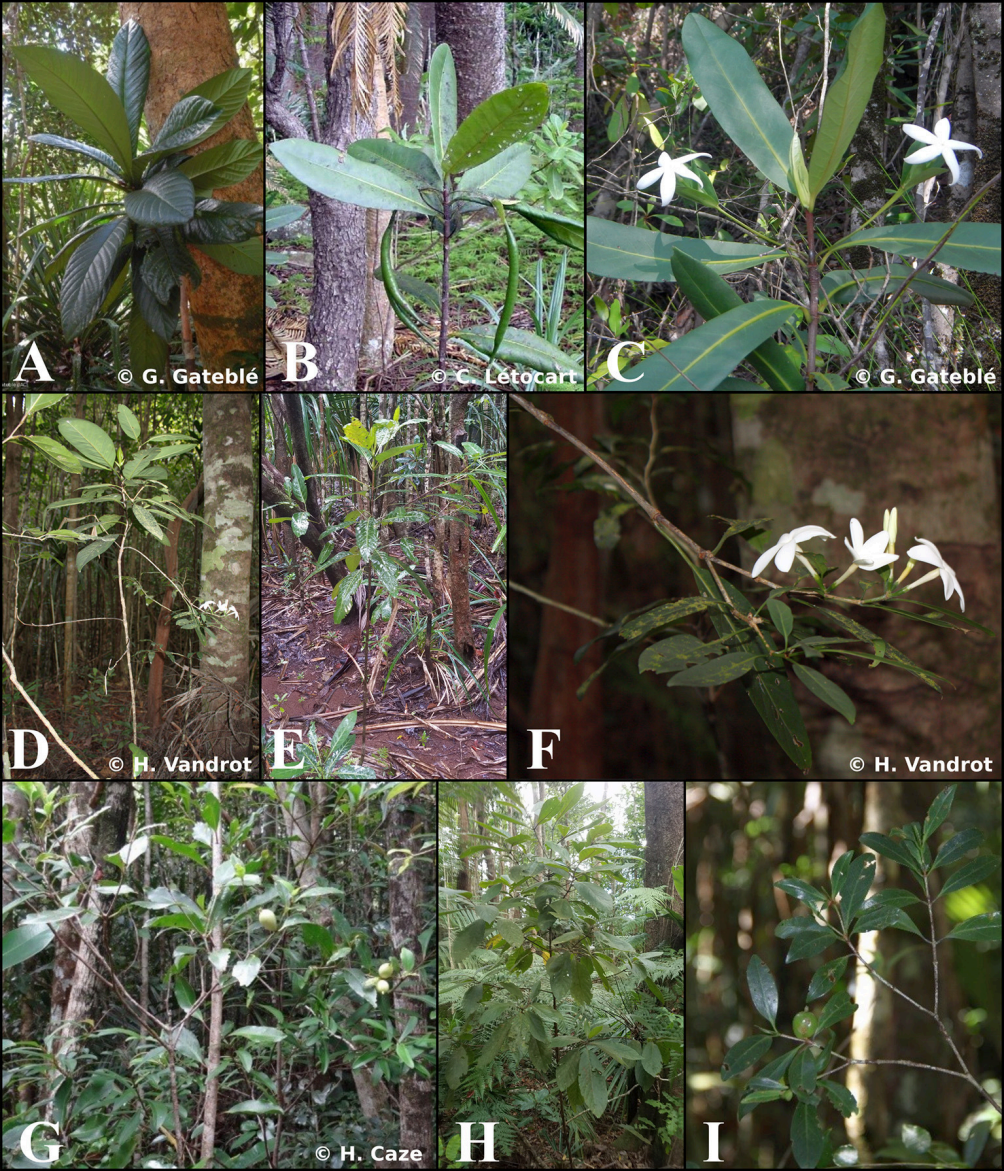
Photographs of different *Atractocarpus* species in their environment showing variability in growth habit and lateral axis. Monocaulous species: **(A)** *A. confertus*, **(B)** *A. bracteatus*, **(C)** *A. bracteatus*. Intermediate species: **(D)** *A. ngoyensis*, **(E)** *A. ngoyensis*, **(F)** *A. ngoyensis*. Branched species: **(G)** *A. pseudoterminalis*, **(H)** *A*. sp. nov. 10, **(I)** *A. pseudoterminalis*.

*Atractocarpus* Schltr. & K. Krause (Gardenieae, Rubiaceae), a Pacific genus of about 40 species, of which 32 are endemics to New Caledonia (Mouly et al., under review), is ideally suited to study the evolution of plant architecture and to test hypotheses regarding the role of architecture in plant ecological diversification. Especially since a recently published phylogeny based on comprehensive sampling is available for the genus (Mouly et al., under review). Most members of this genus are treelets occurring in the understory of tropical and subtropical rainforests, and they exhibit substantial variation in branching pattern (Tirvengadum and Sastre, 1979; Robbrecht and Puff, 1986), ranging from monocaulous (i.e., unbranched) to well-branched species, including various intermediate states (Figure 1). The New Caledonia members of *Atractocarpus*, which originated from a single colonization event that occurred ca. 2.4 Myr ago (Mouly et al., under review), has the highest diversification rate of any genus studied to date (1.17 species species^−1^ Myr^−1^), approaching that of the well-known Hawaiian lobelioid genus *Cyanea* on the island of Maui (1.36 species species^−1^ Myr^−1^, Givnish et al., 2009).

The presence of a high diversification rate combined with architectural diversity is thought to provide an indication of ecological diversification, a situation that is often associated with island colonization (Silvertown, 2004; Silvertown et al., 2005). In groups adapted to growing in the forest understory, light is a variable and limiting resource, and in response, plants have deployed various growth strategies related to leaf photosynthesis and the economization of carbon. To quantify the architectural gradient from monocaulous (unbranched) to well-branched species, we developed a new “functional branching index,” which assesses branching incidence on functional traits that influence assimilation and exploration functions. Considering each understory species of *Atractocarpus* in New Caledonia, we analyzed correlations between architecture and traits associated with key ecologically significant functions (viz., photosynthesis, hydraulic, mechanics, and dispersal). We investigated the evolution of plant architecture (branched vs. unbranched) in forest understory species and its impact on the exceptional diversification rate of the genus. Finally, we explored how ecological opportunities might stimulate the diversification of architecture through spatial differentiation in resource use (niche partitioning) in closely related species following the colonization of New Caledonian rainforests.

## Materials and Methods

### Sampling

We sampled all the 27 known rainforest species of *Atractocarpus* occurring in New Caledonia (list of taxa in Appendix 1 in Supplementary Material). For Ancestral Character Estimation (ACE), three Australian *Atractocarpus* species and one species in each of the most closely related genera for which DNA sequences were available (Mouly et al., 2014, under review) were included as outgroups.

Individuals were sampled in 20 rainforest sites occurring on *Grande Terre*, New Caledonia's main island (Appendix 1 in Supplementary Material).

### Branching Index and Plant Architectural Traits

Based on the observation that an observed architectural gradient might result from differential allocation in branch length and supported leaf area (Figure 1), we used two functional branching indexes based on the differentiation of function from branches to trunk (Corner, 1958). Given that photosynthetic function can be approximated by leaf area (Pérez-Harguindeguy et al., 2013) and exploration function by stem length (Barthélémy and Caraglio, 2007; Smith et al., 2014), branching indexes were calculated as follows:

- Photosynthetic branching index: $\frac{Leaf\;area\;supported\;by\;branches}{Total\;leaf\;area\;(branches+trunk)}$
- Exploration branching index: $\frac{Branches\;cumulated\;length}{Total\;stem\;length\;(branches+trunk)}$

A value of zero indicates that photosynthesis and exploration are assumed only by the trunk and that branches (lateral exploration) are lacking, which corresponds to the monocaulous habit, physiognomically defined as “trees with a single trunk or visible stem of the plant” (Hallé et al., 1978). The higher the value of the index, the greater the functional role played by branches. We built a single integrative branching index that expresses the architectural gradient by combining these two variables using Principal Component Analysis (correlation between photosynthetic and exploration branching indexes: rho = 0.86) employed in the *ade4* package for R (Dray and Dufour, 2007).

Species were segregated into three architectural classes (Monocaulous, Intermediate, and Branched) using Wilcoxon signed-rank tests that enable detection of breaks in the distribution of the integrative branching index.

Using morphological criteria reviewed by Barthélémy and Caraglio (2007), we then described main traits commonly used in architectural analyses (growth process, branching pattern, position of reproductive structures, etc.) for the 27 New Caledonian species of *Atractocarpus*. The number of individuals studied varied from more than a hundred in some species (e.g., *A. pseudoterminalis, A. ngoyensis* and *A. bracteatus*) to 12 in *A. sezitat*, a rare species whose habitat is highly disturbed by introduced herbivores. All species were studied at different ontogenetic stages (from very young plants to senescent adults) except *A. sezitat* and *A*. sp. nov. 12, for which no young plants could be found.

### Plant Functional Traits

We tested the correlation of 14 traits with branching index. The traits were selected to reflect important features of plant ecological strategies (Table 1). They were measured on five individuals per species and, when possible (e.g., for leaf and internode traits), five times per individual. The selection of individuals was standardized for environment (rainforest understory) and for ontogeny following three criteria: (i) recently mature individuals, (ii) non reiterated individuals (see Oldeman, 1974; Barthélémy and Caraglio, 2007), and (iii) non-traumatized trunks. It was not possible to locate individuals meeting all three conditions for *A. sezitat* and *A*. sp. nov. 12 due to small population sizes and habitat degradation, so these taxa were removed from this analysis. We concentrated our sampling in the apical part of the trunk because (i) this enabled standardization of physiological age and (ii) unbranched and branched species can be compared only on the basis of the main stem. Moreover, variation in resource allocation and anatomy between unbranched and branched species might be more prevalent toward the apex, where large leaves are deployed (Carlquist, 1974). Stem and leaf measurements were standardized as follows. Leaf traits (SLA, leaf area) were measured on the five youngest, fully expended leaves of the main axis (trunk). Wood was collected below the terminal leaf tuft for the measurement of anatomical traits (proportion of tissues, specific wood density, and specific stem density). Internode length and diameter were measured on the five youngest, well-developed trunk-internodes (before secondary growth). Branch traits (cumulated length, leaf number, leaf area) were measured on two representative branches per individual and the total number of branches was counted.

**Table 1:** Functional traits measured for 25 *Atractocarpus* species.

**Trait**	**Abbreviation**	**Unit**	**Formula**	**Related function**	**References**
**TRUNK TRAITS**					
Slenderness ratio	sr	Ø	$\frac{Plant\;height}{Plant\;basal\;diameter}$	Geometric feature of buckling resistance	Almeras et al., 2004; Niklas et al., 2006; Homeier et al., 2010
Specific wood density	swd	g.cm^−3^	$\frac{Wood\;dry\;mass}{Wood\;fresh\;volume}$	Growth rate, drought resistance and mechanical reinforcement	Wright et al., 2007; Olson et al., 2009; Pérez-Harguindeguy et al., 2013
Specific stem density	ssd	g.cm^−3^	$\frac{Stem\;dry\;mass}{Stem\;fresh\;volume}$	Growth rate, capacitance, mechanical reinforcement	Pérez-Harguindeguy et al., 2013; Díaz et al., 2016
Internode diameter	internode_d	cm	$\sqrt{Min\;IN\;diameter\times Max\;IN\;diameter}$	Growth rate, mechanical reinforcement	Vertessy et al., 1995; Schuerger et al., 1997; Olson et al., 2009
Internode length	internode_l	cm	Ø	Growth rate, leaf spacing	Weijschedé et al., 2007; Dong et al., 2010
Proportion of pith	%_pith	Ø	$\frac{Area\;of\;pith}{whole\;section\;area}$	Primary growth, stem biomechanics	Carlquist, 1974; Niklas, 1992; Levionnois et al., 2018
Proportion of wood	%_wood	Ø	$\frac{Area\;of\;wood}{whole\;section\;area}$	Secondary growth, stem biomechanics	Penfound, 1931; Schuerger et al., 1997; Hummel et al., 2007
Proportion of cortex	%_cortex	Ø	$\frac{Area\;of\;cortex}{whole\;section\;area}$	Storage	Pérez-Harguindeguy et al., 2013
Huber value	hv	Ø	$\frac{Wood\;area\;at\;POM}{LF\;area\;above\;the\;POM}$	Allocation of wood to leaf area deployed	Penfound, 1931; Gleason et al., 2012; Pérez-Harguindeguy et al., 2013
Mean leaf area	mean_la	cm2	Ø	Energy balance, hydraulic demand	Ackerly, 2004; Poorter et al., 2009; Pérez-Harguindeguy et al., 2013
Specific Leaf Area	sla	cm.g^−1^	$\frac{Leaf\;fresh\;area}{Leaf\;dry\;mass}$	Allocation of biomass to light harvesting	Westoby et al., 2002; Pérez-Harguindeguy et al., 2013
**WHOLE PLANT TRAIT**					
Total leaf area	total_la	cm2	(*BR mean LF area* × *Number of BR*) + (*Mean area of TR LF* × *Number of TRLF*)	Energy balance, hydraulic demand	Pérez-Harguindeguy et al., 2013; Díaz et al., 2016
Leaf number	nb_leaf	Ø	*Number of TR LF* + (*BR mean number of LF* × *Number of BR*)	Light harvesting	Duncan and Hesketh, 1968; White, 1983a
**FRUIT TRAIT (HERBARIUM MEASURE)**					
Fruit volume	fruit_vol	cm^3^	*Core fruit length* × (*Core fruit width*)^2^	Dispersal, reproductive allocation	Cornelissen, 1999; Cornelissen et al., 2003

*References are given to justify the interest of each trait for corresponding functions. IN, internode; POM, point of measurement; LF, leaf; BR, branch; TR, trunk*.

Most of the individuals studied in the field were infertile, so fruit traits were measured on herbarium specimens [NOU and P; acronyms follow Thiers (continuously updated)]. We selected undamaged and unflattened fruits from which length and diameter were measured. As *Atractocarpus* fruits are often ellipsoid to tubular (Puttock, 1999), these two dimensions are used to approximate fruit volume (Table 1). A total of 592 fruits were measured, and only the five largest fruits per taxon (four for *A. confertus* and *A. sessilifolius*) were used to ensure that values from only mature fruits were included in our analyses. One of the species, *A*. sp. nov. 6, is a very rare species whose fruits have never been observed; it was consequently excluded from the fruit volume dataset.

### Data Analysis

#### Phylogenetic Tree

The molecular phylogeny of Mouly et al. (under review) was used for analysis. This ultrametric phylogenetic tree was constructed under Bayesian Inference using one nuclear (*ITS*) and two chloroplastic (*trnTL, rpl32*) loci. Since the current study focused on rainforest species, we pruned the five dry forest and maquis species using the R software (Ver. 3.4.3).

#### Phylogenetic Signal

We assessed phylogenetic independence of measured traits (Felsenstein, 1985) using two complementary statistics calculated under a Brownian Motion (BM) model of evolution. Pagel's Lambda (Pagel, 1999; Freckleton et al., 2002) is widely used for low rates of type I error and robustness, even for poorly or moderately informative phylogenies (Freckleton et al., 2002; Münkemüller et al., 2012). Because this statistic loses statistical power when used on small phylogenies (<30 terminals, Freckleton et al., 2002), we also used Blomberg's Kappa (Blomberg et al., 2003; Kembel, 2009) that is less robust but more appropriate for small taxonomic sampling (until 20 taxa, Kamilar and Cooper, 2013). Both statistics were calculated considering intraspecific variation using the *phytools* package for R (Revell, 2012).

#### Trait Correlations and Functional Characterization

We used phylogenetic regression based on the method proposed by Ives et al. (2007) and implemented in the *phytools* package for R (Revell, 2012) to test whether architectural variation was correlated with functional specialization. Using maximum likelihood, this method fits bivariate models taking into account both phylogenetic framework and intraspecific variation. The response variable was the integrative branching index, and the explanatory variables were all other functional traits (Table 1). Significance of relations was tested using a likelihood ratio test between the model and a model constrained with a slope of zero.

To characterize the functional space of each architectural classes, we performed a Principal Component Analysis. We used the species arithmetical mean of each functional traits (branching index excluded), and functional differences between architectural classes (Monocaulous, Intermediate, Branched) were tested with a permanova (*Vegan* package for R, Oksanen et al., 2018).

#### Ancestral Character Estimation

To determine the putative ancestral architectural class of *Atractocarpus* and infer the evolution of architecture in the genus, Ancestral Character Estimation (ACE) was performed. We assigned an architectural class to each of the 11 outgroup species based on published descriptions (Fosberg, 1987; Smith and Darwin, 1988; Fosberg et al., 1993; Puttock, 1999; Wong, 2004; Zahid and Wong, 2004, 2010; Tong et al., 2013), herbaria specimens (P, K, BM, E) and available photos. The ACE were performed using a maximum likelihood method under the *ape* package for R (Paradis et al., 2004). Three possible models of evolution fitted the data characteristics: (i) equal transition rates between classes (ER), (ii) different transition rates between classes but with equal rates for reversions (SYM), and (iii) different rates for every transition (ARD). The best model was selected using the corrected Akaike Information Criterion (AICc).

#### Trait Based Diversification

To test whether one of the architectural classes has contributed more than the others to the diversification of New Caledonian *Atractocarpus* (by increasing speciation rates and/or decreasing extinction rates), we used the Multiple State Speciation Extinction (MuSSE) framework (Fitzjohn et al., 2009) as implemented in the *diversitree* package for R (Fitzjohn, 2012). For this analysis, outgroups were dropped from the phylogeny to consider only the 27 rainforest *Atractocarpus* species. Fifteen models of diversification were used, each differing in whether or not of speciation, extinction and transition rates were equal between classes (Table 2). Model selection was done according to AICc.

**Table 2:** Models fitted for trait based diversification analysis.

**Model**	**Speciation rates**	**Extinction rates**	**Transition rates**	**AICc**
1	≠	≠	≠	−46.72
**2**	=	=	=	−**50.59**
3	≠	=	=	−49.59
4	≠	≠	=	−49.56
5	≠	=	≠	−46.72
6	=	=	≠	−46.16
7	=	≠	≠	−46.16
8a	M = I; B ≠	=	=	−50.58
8b	I = B; M ≠	=	=	−50.50
8c	M = B; I ≠	=	=	−49.59
9a	M = I; B ≠	0	=	−50.58
9b	I = B; M ≠	0	=	−50.50
9c	M = B; I ≠	0	=	−49.59
**10**	=	**0**	=	−**50.59**
11	≠	0	=	−49.59

*Bold lines represent models with lowest AICc. M, Monocaulous; I, Intermediate; B, branched*.

## Results

### Branching Index

**Figure 2:**
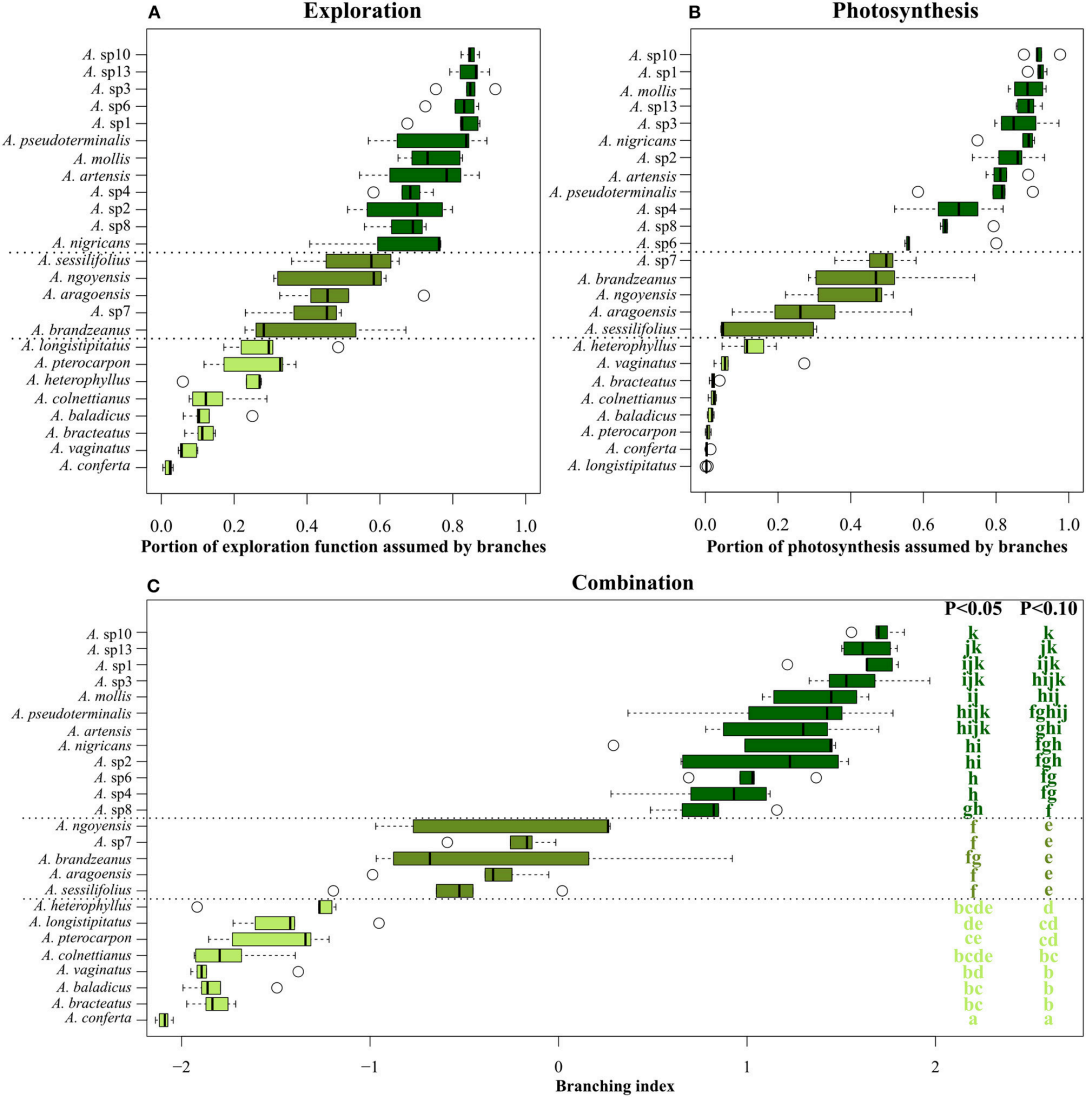
Branching indexes calculated on the base of **(A)** exploration function, **(B)** photosynthetic function and **(C)** the combination of both, for 25 *Atractocarpus* species. Letters in **(C)** correspond to the result of the Wilcoxon test; species with shared letters are not significantly different for a given risk of error.

Branching indexes confirmed the existence of a morpho-functional gradient from branched to monocaulous species of *Atractocarpus*, for both exploration and photosynthetic functions (Figures 2A,B). For both indexes, interspecific variation was too gradual to allow the partitioning in distinct classes, but when combined (i.e., using the branching index), three distinct architectural groups could be differentiated (Wilcoxon tests: *P* < 0.1, Figure 2C), viz. the branched, intermediate and monocaulous architectural classes. On average, branched species had 82% of photosynthesis and 76% of exploration provided by branches, vs. 36 and 45%, respectively, for intermediate species, and 3 and 16% for monocaulous species.

### Architectural Characterization

**Figure 3:**
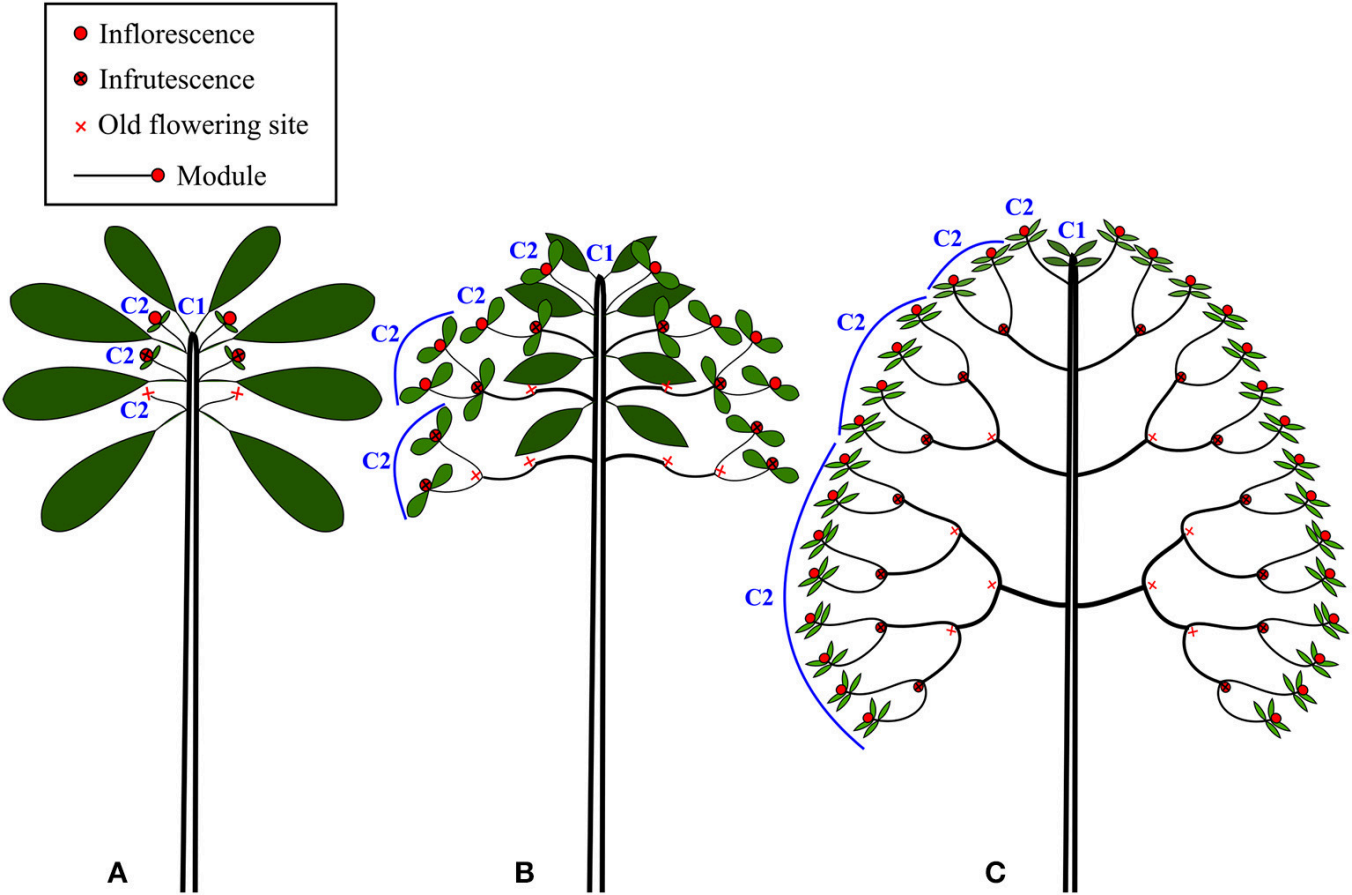
Schematic representation of the three main architectural classes found in the rainforest understory species of *Atractocarpus*: **(A)** Monocaulous, **(B)** Intermediate **(C)** Branched.

**Figure 4:**
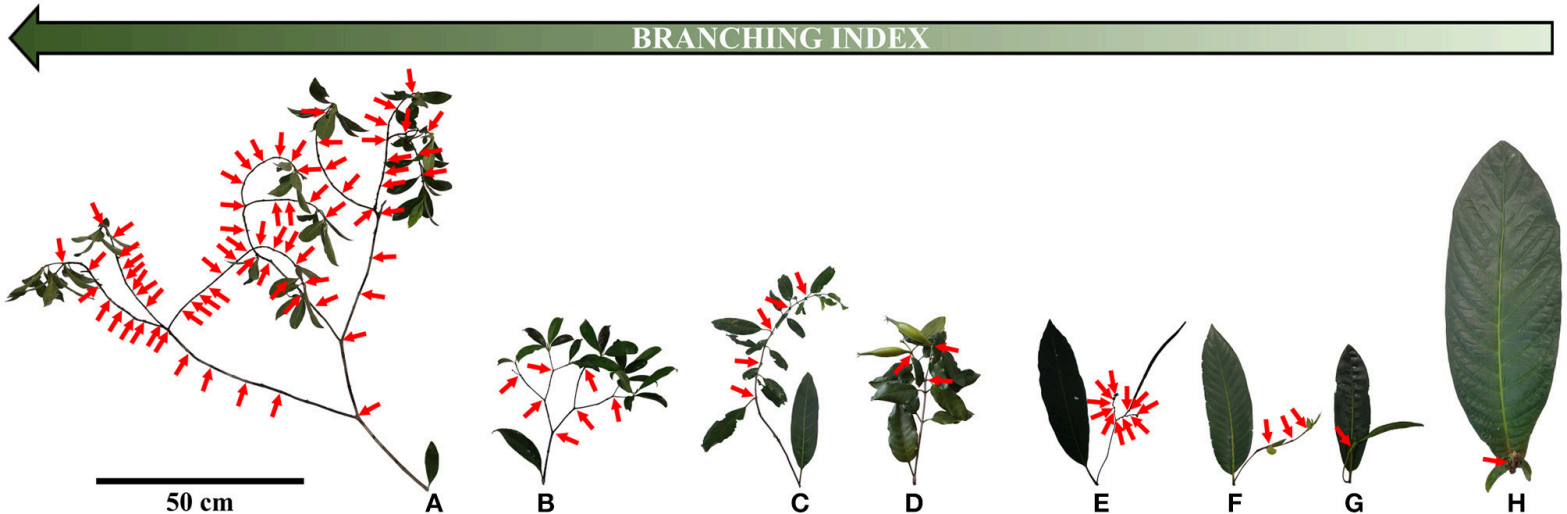
Photographs of branches with their axillary leaves (i.e., from trunk) for height *Atractocarpus* species with different branching degrees. Arrows indicate apical death, i.e., flowering sites (for *A. longistipitatus* **(E)**, only half of apical death has been represented). Branched species: **(A)** *A. pseudoterminalis*, **(B)** *A*. sp4. Intermediate species: **(C)** *A. ngoyensis*, **(D)** *A. brandzeanus*. Monocaulous species: **(E)** *A. longistipitatus*, **(F)** *A. pterocarpon*, **(G)** *A. bracteatus*, **(H)** *A. confertus*.

The architecture of New Caledonian *Atractocarpus* species involves two axis categories: (i) a trunk (C1), i.e., an orthotropic monopodium with continuous indeterminate growth, and (ii) “branches” (C2), i.e., orthotropic sympodia with terminal sexuality conferring rhythmic growth (Table 3, Figure 3). The sympodial branching of C2 always originates in a hypotonic (i.e., on the lower surface) or amphitonic position, resulting in upward flower exposure (Figure 1). The only qualitative architectural variation observed between species concerned the branching position of C1, which was continuous for some monocaulous species while diffuse for others. Two main quantitative architectural traits varied greatly among species, the number of modules per branch and the length of modules, which decreased in monocaulous species (Table 3, Figures 3, 4). For example, *A. longistipitatus* is a monocaulous species whose C2 comprise many very short modules (apart from the first one). By contrast, *A. bracteatus* is also monocaulous but its C2 comprise a single long module (Figure 4). The number of internodes per module vary little in the genus but tend to be reduced in monocaulous species, with an extreme case of one node per module in *A. confertus* (Table 3). All species showed the ability to develop delayed reiterate on damaged or aged individuals.

**Table 3:** Architectural traits of 27 *Atractocarpus* species.

**Taxon**		***A. mollis***	***A*. sp10**	***A*. sp8**	***A*. sp1**	***A*. sp6**	***A*. sp13**	***A*. sp3**	***A. nigricans***	***A. pseudoterminalis***	***A. sezitat***	***A*. sp4**	***A*. sp12**	***A. artensis***	***A*. sp2**	***A. aragoensis***	***A. ngoyensis***	***A. brandzeanus***	***A*. sp7**	***A. sessilifolius***	***A. longistipitatus***	***A. baladicus***	***A. bracteatus***	***A. pterocarpon***	***A. heterophyllus***	***A. colnettianus***	***A. vaginatus***	***A. conferta***
Architectural class		Branched														Intermediate					Monocaulous							
Number of AC		2														2					2							
C1	Growth pattern	Indeterminate														Indeterminate					Indeterminate							
	Growth periodicity	Continuous														Continuous					Continuous							
	Branching pattern	Monopodial														Monopodial					Monopodial							
	Growth direction	Orthotropic														Orthotropic					Orthotropic							
	Reproduction	Absent														Absent					Absent							
	Branching position	Diffuse														Diffuse					Diffuse			Continuous				
C2	Growth pattern	Determinate														Determinate					Determinate							
	Growth periodicity	Rhythmic														Rhythmic					Rhythmic							
	Growth direction	Orthotropic														Orthotropic					Orthotropic							
	Reproduction	Terminal														Terminal					Terminal							
	Branching position	Rhythmic acrotone														Rhythmic acrotone					Rhythmic acrotone				na			
	Branching pattern	Sympodial														Sympodial					Sympodial				na			
	Number of relay	1-2														1-2					1-2				na			
	Number of internode/module	2 (-3)								2-3						2-3			2 (-3)	2	2				2			1
	Max. number of module/branch	56	48	46	42	41	38	38	31	71	48	30	29	28	25	25	19	12	16	46	26	5	5	9	5	1	1	1
	Mean length of module (cm)	5.5	4.8	6.5	8.9	9.2	7.8	7.6	7	5.9	6.9	6.5	7.7	14.8	11.5	6.3	12.9	15	7.1	2.4	1.5	12.1	8.1	4.5	8.5	5.9	5.1	1.7

*AC, Axis category; C1, First axis category (trunk); C2, Second axis category (“branches”)*.

### Functional Characterization

Phylogenetic signal varied substantially depending on which statistic was used (Lambda or Kappa, Table 4). This was probably due to the small size of our phylogeny (25 terminals), since the statistics differ in sensitivity to the number of terminals (Freckleton et al., 2002; Kamilar and Cooper, 2013). Blomberg's Kappa captures significant phylogenetic signal for stem specific density, proportion of pith, Huber value, and mean leaf area, whereas Pagel's Lambda captures significant signal for internode diameter (Table 4). Among all measured functional traits, only the branching index had a highly significant phylogenetic signal calculated with both Pagel's Lambda and Blomberg's Kappa.

**Table 4:** Phylogenetic signals of measured traits across the phylogeny of 25 *Atractocarpus* species (24 for fruit volume).

**Trait**	**Lambda**	***p*-value**	**Kappa**	***p*-value**
**Branching index**	**1.013**	<**0.001**	**1.132**	**0.002**
Slenderness ratio	0.748	1	0.251	0.539
Specific wood density	0.447	1	0.429	0.242
Specific stem density	0.813	0.265	**0.607**	**0.029**
Internode diameter	**0.442**	**0.047**	0.533	0.076
Internode length	0	1	0.127	0.946
Proportion of pith	0.277	0.427	**0.603**	**0.021**
Proportion of wood	0.595	1	0.399	0.39
Proportion of cortex	0.488	1	0.418	0.27
Huber Value	0.589	0.998	<**0.001**	**0.035**
				
Total leaf area	0.765	1	0.006	0.165
Leaf number	1.105	1	0.001	0.157
Mean leaf area	1.105	0.064	**0.031**	**0.003**
Specific Leaf Area	0	1	0.042	0.405
Fruit volume	0	1	0.002	0.356

*Signal were tested with Pagel's Lambda (left) and Blomberg's Kappa (right). Bold lines represent significant phylogenetic signal following a 0.05 risk of error*.

**Figure 5:**
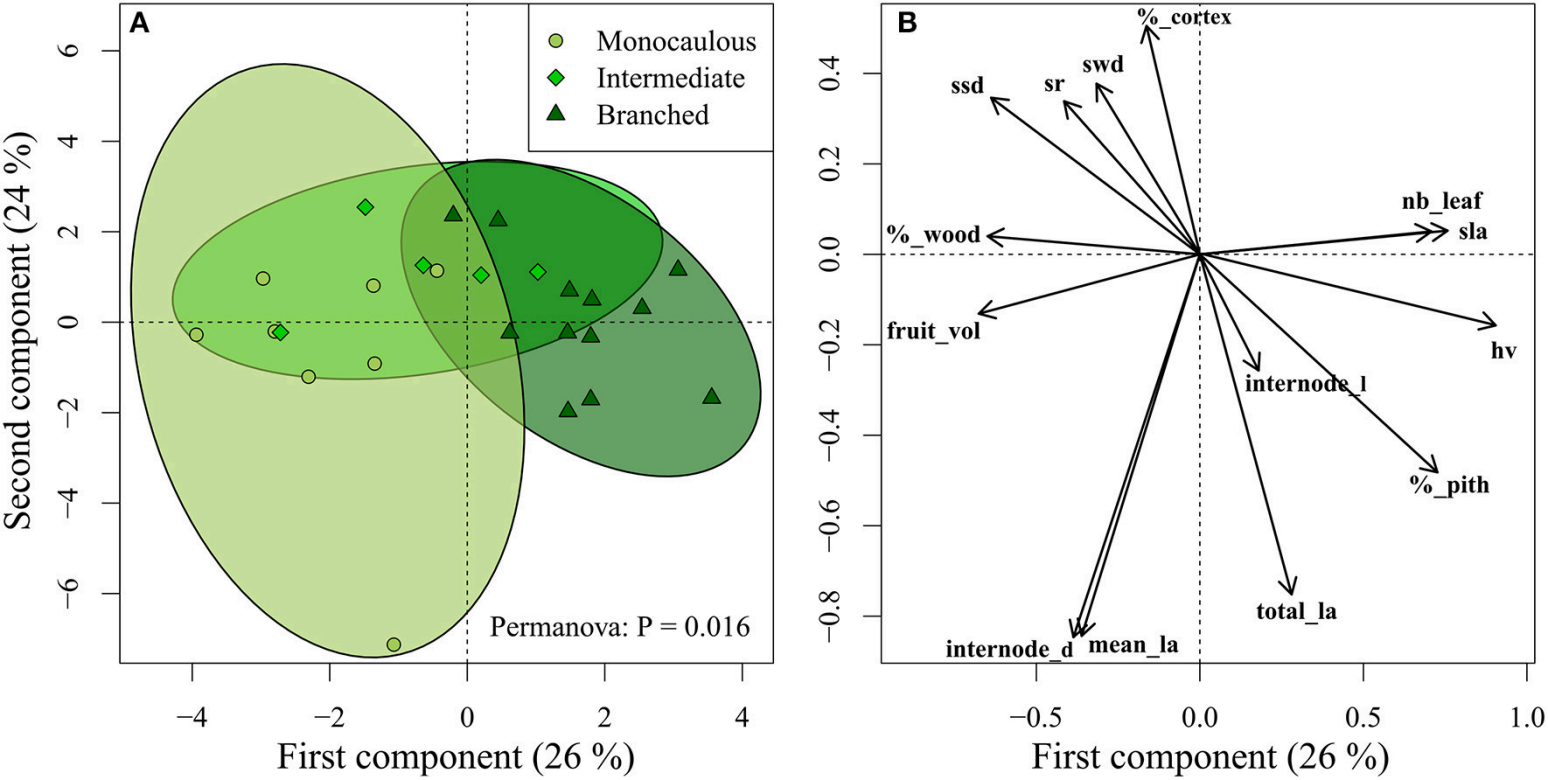
Projection of **(A)** species and **(B)** traits on the two first axis of Principal Component Analysis (see Table 1 for trait abbreviations). Ellipses represent the 95% confidence interval for each architectural classes. Functional differences between architectural classes were tested with Permanova.

Several functional traits were significantly related to the branching index (Table 5), including biomechanics (e.g., slenderness ratio, internode diameter) as well as photosynthesis (e.g., leaf area, SLA), hydraulics (e.g., Huber value), and even dispersal (fruit volume). These correlations between architecture and functions were confirmed by PCA and Permanova (*P* = 0.016), which showed significant differences between the functional space occupied by the three architectural classes (Figure 5). Intermediate species were confounded between the two other architectural classes, but monocaulous and branched species appeared to be functionally very different. In comparison to monocaulous species, branched taxa tend to have numerous small leaves, higher SLA, smaller fruits and, smaller internode diameters with a lower proportion of wood, but a higher wood area-leaf area ratio (Huber value). The monocaulous *A. confertus* (the lowest point on Figure 5) was distinct from the other *Atractocarpus* species examined in having numerous very large leaves, leading to a disproportionately higher total leaf area (up to 2.4 m2 vs. a mean of 0.5 m2 for the other species).

**Table 5:** Results of phylogenetical generalized least square.

**Trait**	**log(L)**	**Beta**	***p*-value**
**Slenderness ratio**	−**150.3**	−**3.1**	**0.004**
Specific wood density	−0.635	−0.003	0.157
Specific stem density	4.982	−0.0002	0.290
**Internode diameter**	−**35.90**	−**0.09**	**0.018**
Internode length	−85.30	0.3	0.169
Proportion of pith	0.397	0.03	0.112
**Proportion of wood**	**1.477**	−**0.0009**	**0.002**
Proportion of cortex	−2.600	−0.01	0.094
**Huber value**	−**295.9**	**3587**	<**0.001**
Total leaf area	−282.5	288.8	0.351
**Leaf number**	−**165.2**	**37.2**	<**0.001**
**Mean leaf area**	−**203.3**	−**85.6**	**0.008**
**Specific Leaf Area**	−**162.9**	**15.9**	<**0.001**
**Fruit volume**	−**180.5**	−**3.8**	**0.021**

*Bold lines represent significant relation between corresponding trait and branching index following a 0.05 risk of error*.

### Ancestral Character Estimation

**Figure 6:**
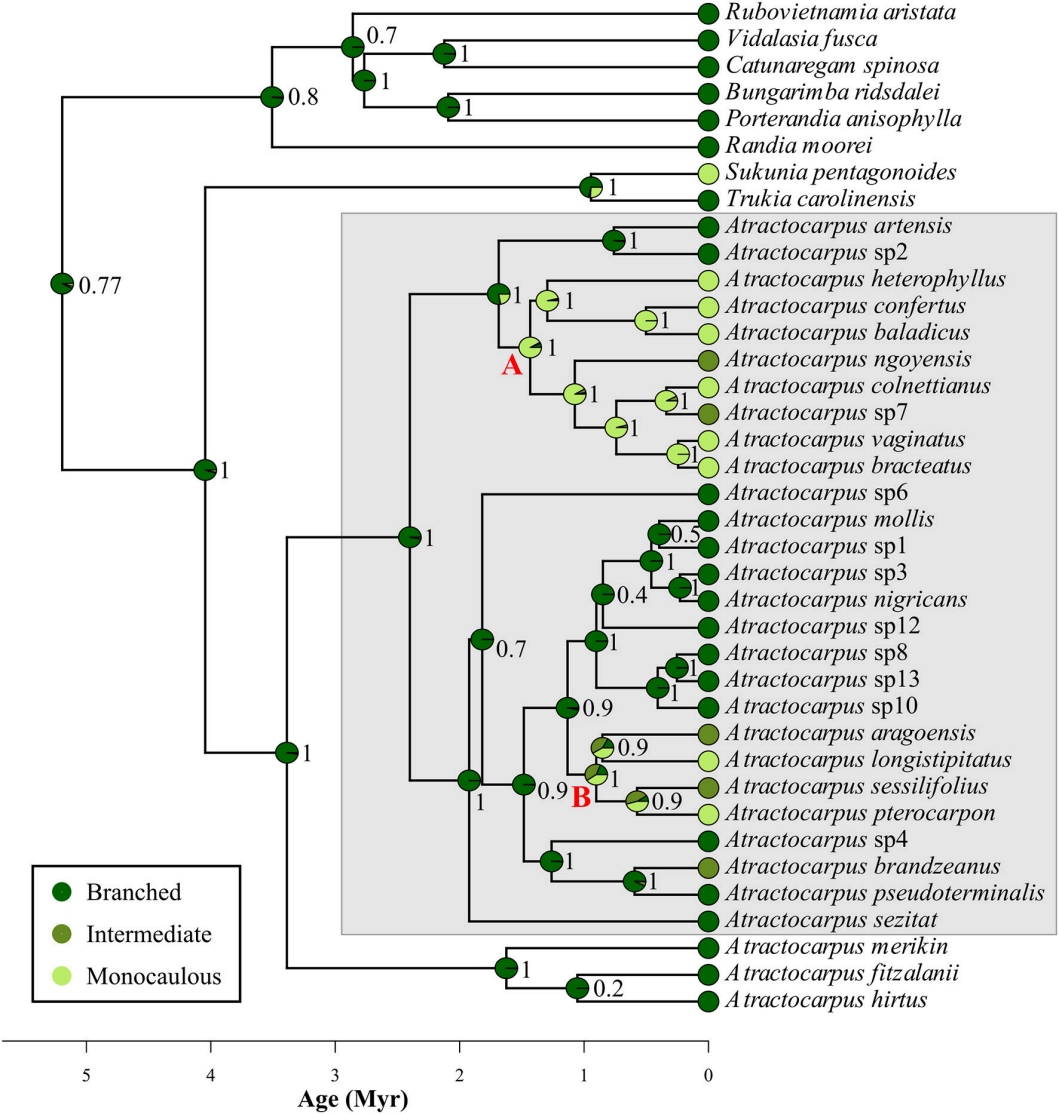
Estimation of ancestral architectural class in the genus *Atractocarpus*. Numbers correspond to the Bayesian probability for each reconstituted node. Gray box highlights New Caledonian clade. Letters (A, B) highlight major clades were shifts from branched to monocaulous or intermediate architecture operate.

All considered outgroup species belonged to the branched architectural class, except for the Fijian *Sukunia pentagonoides* (Seem.) A.C.Sm, which is monocaulous. Among the three possible models of evolution that fitted the data, the best supported one was that of equal transition rates between classes (ER), with an AICc of 84.4 vs. 96.1 and 104.2 for SYM and ARD, respectively. According to this model, the ancestral architecture of *Atractocarpus* was most likely branched (probability = 0.98, Figure 6), and the monocaulous habit evolved two or three times in the two main New Caledonian clades (referred to as “monocaulous clade A” and “monocaulous clade B” in Figure 6), ca. 1.4 and 0.9–0.6 Myr ago respectively. In each clade, species with an intermediate architecture are closely related to monocaulous species, the only exception being *A. brandzeanus*, whose closest relatives are branched. Conversely, pairs of sister or closely related species never showed branched and monocaulous habit.

### Trait Based Diversification

Following the IACc, models 2 and 10 are the best fitted, in which speciation and transition rates are equal for each state and extinction rates are equal or null (Table 2). This means that diversification was not greater among the members of any of the architectural classes during the evolution of *Atractocarpus* in New Caledonia.

## Discussion

### Evolution of Plant Architecture

Despite the diversity of growth habits in the genus *Atractocarpus*, ranging from well branched to monocaulous species, the architectural construction of members of the group is fairly homogenous. Differences between species mainly result from quantitative variations in which the number of modules per branches and module length tend to increase with increasing branching index.

The measurement of functionally explicit variables (branch length and leaf area) that link plant architecture and function (Lauri, 1988; Lauri and Kelner, 2001; Charles-Dominique et al., 2017) showed that variation of growth habit in *Atractocarpus* is correlated with a gradual variation from branched species, for which photosynthesis and exploration are largely assumed by branches, toward monocaulous species, for which these functions are assumed by the trunk. The reduced C2 in monocaulous species supports a limited number of small leaves (on average 4 leaves of 9 cm^2^) and are dedicated almost exclusively to reproduction. These “axes” are functionally closer to inflorescences than branches (Van Steenis, 1963) and are often considered as such by taxonomists (e.g., Fosberg et al., 1993; Puttock, 1999).

The branched architectural class appears to be ancestral in *Atractocarpus* and our results indicated at least two independent origins of monocauly in New Caledonia. Other examples of derived monocauly have recently been revealed in flowering plants (Chomicki et al., 2017; Barrabé et al., 2018). The intermediate architectural class is evolutionary closer to the monocaulous habit since intermediate and monocaulous species are always sister taxa or very close relatives. There is a high probability that the putative ancestor of “monocaulous clade B,” had an intermediate architecture. This insight, as well as the architectural gradient observed among extant species, suggests a gradual evolutionary transition from the branched architecture toward monocauly through an intermediate architectural class. Reversion from a monocaulous architecture toward an intermediate one appears to have been possible (e.g., *Atractocarpus* sp. nov. 7) but full reversion to a branched architecture was not observed.

The similarity of developmental origin and the gradual quantitative architectural variation of lateral axes (C2) across closely related taxa suggest a homologous origin of these axes in the *Atractocarpus* species studied here (see Nozeran, 1955; Rossignol and Rossignol, 1985; Haicour et al., 1989). A homologous origin between vegetative and inflorescence axes has been suspected in several groups such as Ranunculaceae and Papaveraceae (Stebbins, 1973), as well as several families of Alismatales (Posluszny and Charlton, 1993, 1999) and Pandanales (Rudall and Bateman, 2006), though none of these studies combined architectural analysis with molecular phylogenetic results.

Only a few studies to date have explored evolutionary links between architectural models (see Chomicki et al., 2017). In New Caledonia *Atractocarpus*, we found recurrent transitions from Stone's model for branched species (with C2 functioning as branches) to Corner's model for monocaulous species (with C2 functioning as inflorescences), although the limit between them remains fuzzy, as illustrated by species whose architecture is intermediate. The variation observed in branching degree across species illustrates an “architectural continuum” (Oldeman, 1974; Barthélémy et al., 1989) rather than saltational evolution involving rapid and profound change in architecture (Bateman and DiMichele, 1994; Bateman, 1999). Nothing suggests that the intermediate architectural class confers lower fitness (i.e., represents a fitness valley) compared to monocaulous or branched architecture, particularly in situations where the environment (rainforest understory) varies gradually. Saltation from one model to another might take place when gradual changes are impossible, for example in the colonization of contrasted habitats (Bateman and DiMichele, 1994). Gradual variation between and within models has already been noted (Hallé et al., 1978), suggesting that preferential transition between models might occur.

The two independent origins of monocauly inferred in *Atractocarpus* illustrate two different evolutionary pathways involved in the transition from branches into inflorescences. In “monocaulous clade A,” reduction in the branching index occurred through a reduction in the number of modules, while in “monocaulous clade B,” module length is involved. Architectural analysis actually showed that the two species with intermediate architecture and the two monocaulous species belonging to the clade B are those with the higher number of modules (up to 46 for the intermediate *A. sessilifolius* and 26 the for monocaulous *A. longistipitatus*, i.e., as much as certain branched species) and with shorter module length (excluding the monocaulous *A. confertus*). This type of morphological reduction observed at the module level in *Atractocarpus* could affect any elementary level of organization in other plant groups (e.g., annual shoots, growth units, metamers, etc.).

The iterative gradual transition of axillary structure (C2) from branches to inflorescences occurs as a functional reduction of vegetative growth and a change in the timing of flowering. Such evolutionary changes, often result from growth heterochrony (Smith, 2001). In the case of New Caledonian *Atractocarpus*, the axillary structure (C2) of descendants is either a truncated part (in “monocaulous clade A”) or a miniaturized copy (in “monocaulous clade B”) of that of their ancestors. This suggests heterochronic evolution through “neoteny” in the first case and through “proportional dwarfism” in the second (Gould, 1977; Alberch et al., 1979; Smith, 2001), but more details of the ontogenetic sequences are required to test these hypotheses in *Atractocarpus*.

In this context, our original approach showed that a careful study of low organization levels is needed to depict the evolution of plant architecture. This involves a deep characterization of architectural units (Edelin, 1977, 1984), combining qualitative and quantitative traits.

### Branching Index, Corner's Rules, and Ecological Strategies

In the original *Durian theory*, Corner (1949, 1953-1954) proposed two fundamental rules governing plant morphology: (i) *Axial conformity*, stipulating that “the stouter, or more massive, the axis in a given species, the larger and more complicated its appendages”; and (ii) *Diminution on ramification*, stipulating that “the greater the ramification, the smaller become the branches and their appendages”. *Axial conformity* is by far the most studied since the leaf size—twig size and fruit size—leaf size spectra are among the best-documented (White, 1983b; Bond and Midgley, 1988; Brouat et al., 1998; Cornelissen, 1999; Brouat and McKey, 2001; Westoby et al., 2002; Preston and Ackerly, 2003; Westoby and Wright, 2003; Pickup et al., 2005; Sun et al., 2006; Normand et al., 2008). Large leaves and fruit carried by a large single stem, as seen in monocaulous species, illustrate one extremity of this spectrum. The second rule (*diminution of ramification*), has received much less consideration (but see White, 1983a; Ackerly and Donoghue, 1998). Our results agree with this statement, as we have shown that internode diameter, as well as fruit and leaf size, are negatively correlated with branching index, even with phylogenetic corrections. Beyond Corner's rules, we found a strong correlation between branching index and several functional traits related to various ecological strategies in areas with a similar habitat (i.e., rainforest).

We found that branching index tends to be correlated with ecologically important dimensions (Mooney, 1972; Grime, 1974; Grime et al., 1988; Westoby et al., 2002). The large leaves of monocaulous species increase light interception in shady understory (Poorter, 1999; Rozendaal et al., 2006), their low SLA and Huber value are likely associated with low photosynthetic capacity (Field and Mooney, 1986; Shipley et al., 2005; Poorter and Bongers, 2006; Rozendaal et al., 2006; Pérez-Harguindeguy et al., 2013) and their small internode length can result from a slow growth rate (Mooney, 1972; Chuah, 1977; Grime et al., 1988; Westoby et al., 2002). Monocaulous species are likely skewed toward a high resource conservation strategy (Grime et al., 1997; Díaz et al., 2004, 2016) suited to the shady understory of rainforest.

In sparsely branched to unbranched species, a distal part of the stem with thicker diameter and higher wood proportion was found to be suited to supporting large and numerous energetically costly leaves (higher leaf area and SLA). However, higher wood area does not fully compensate for high total leaf area of the main stem since the Huber value (the ratio of wood area to leaf area) was positively correlated with branching index. Detailed anatomical studies, particularly on vessel size and density, are needed to understand the hydraulic and mechanical trade-off involved in the pervasive link between leaf area, stem thickness and branching degree (Lehnebach et al., 2018).

### Divergence and Ecological Opportunities in New Caledonian Rainforests

New Caledonian *Atractocarpus* appear to be the result of a single colonization event (Mouly et al., under review) and the archipelago is the center of diversity for the genus. Island colonization is a two steps process involving dispersal and successful establishment (Silvertown, 2004). The large, fleshy fruits of *Atractocarpus* suggest a rather limited ability for long-distance dispersal. Such niche preemption, claimed to act as a major driver of monophyly and diversification in island floras (Silvertown, 2004), could have prevailed in the diversification of the New Caledonian clade of *Atractocarpus*. Its young age (estimated at 2.4 My) coincides with colonization during late Pleistocene, a period of intense climatic Fluctuation (Bowler et al., 1976; Hope and Pask, 1998; Stevenson and Hope, 2005). Increasing evidence supports the persistence of taxa in rainforest refugia during the Pleistocene in New Caledonia (Pintaud et al., 2001; Pillon, 2012; Nattier et al., 2013; Poncet et al., 2013; Pouteau et al., 2015; Tournebize et al., 2017). Climatic fluctuations causing expansion and contraction of rainforests could also have offered ecological opportunities for new colonizers. Other diversified rainforest clades in New Caledonia have been shown to result from recent colonization (e.g., palms, Pintaud et al., 2001; Pillon, 2012). Similarities in form and physiology among close relatives, as suggested by our character state reconstruction and architectural analysis, are suggested to involve intense competition that favors divergence among locally coexisting species (Givnish, 2016). In support of this, we found notable variation in module length and number between sisters species, even when they belong to the same architectural class. In *Atractocarpus*, variation in growth habit is correlated with a gradual switch in assimilation function from branches to trunk, and is associated with a vertical differentiation of major functions. Leaf arrangement in monocaulous species results in important overlap that impacts photosynthesis, while stem slenderness suggests a rather small structural investment in vertical support. Branched species tend to increase light harvesting and reduce leaf overlap (via increase branch length), a strategy that require more investment in stem tissue (Givnish, 1995), as allocation to non-leaf structures might increase with ramification. *Atractocarpus* species thus exhibit a gradient of foraging for light in which leaf size and disposition vary with the degree of ramification.

New Caledonian rainforests are characterized by a low canopy (ca. 20 m) and trees with small crowns (Blanchard et al., 2016), two structural features that increase the number and intensity of sunflecks (Chazdon and Pearcy, 1991). A low canopy and its corollary, reduced forest stratification, could result in stronger competition within a given stratum. These forest features (high sunfleck variability, reduced stratification) favor niche partitioning through a gradient in architecture and related functional traits. Moreover, limited dispersal of the large fruits of *Atractocarpus* might interact with niche partitioning to promote divergent evolution. Finally, divergent selection may be especially favored in permissive environments where competition prevails over the external environment or in the colonization of islands where resources are underutilized (Givnish, 2016). New Caledonia exhibits both components of divergent selection: (i) climatically permissive rainforests and (ii) ecological opportunities offered by recent climatic fluctuations. Divergent selection, caused by competition among closely related taxa, leads to adaptive radiation, i.e., the rise of a diversity of ecological roles and related adaptations in different species among a lineage (Givnish, 2016). The theory of niche pre-emption holds that adaptive radiation creates a barrier that inhibits the establishment of closely related taxa, thus reducing the likelihood of repeated colonization.

## Conclusion

Despite the fundamental importance of plant architecture, little work has been done to integrate this aspect into key domains of plant science such as evolution and functional ecology. However, increasing availability of information on ecologically important traits and molecular phylogenies provides a basis for testing and developing new concepts. Our study clearly highlights evolutionary processes behind architectural transitions and their link to plant ecological strategy and perhaps also to diversification. We have shown that gradual transition from one architecture to a very different one is possible through morpho-functional reduction of morphological units. Quantifying plant architecture through functional indexes appears to offer a promising avenue toward further understand the implications of architectural variation on plant fitness under different environmental conditions. Based on such an index, our study provides a functional definition of monocauly that is ecologically and evolutionary more explicit than one based solely on physiognomy (cf. Hallé et al., 1978; Chomicki et al., 2017): we define monocaulous species as woody plants whose cardinal functions (e.g., assimilation and exploration) rely on a single apparent stem.

Although our work focused on a small clade, it opens new perspectives and proposes a general framework for further understanding evolution of plant architecture and its functional implications in other plant groups and other geographical areas, and at larger scales. We believe that Pleistocene climatic fluctuations have played a major role in the evolution of monocauly and more widely in shaping the current diversity of the New Caledonian flora. This hypothesis now needs to be tested at a larger phylogenetic scale in New Caledonia, as the monocaulous habit, which occurs repetitively in many different groups (Veillon, 1976; Schmid, 1979, 1990), has much to offer to understand the evolution, biogeography and ecology of this “very old Darwinian island” (Grandcolas et al., 2008).

## Data Availability Statement

All datasets generated for this study are included in the manuscript and the supplementary files. Data concerning the phylogenetic tree was obtained from Mouly et al. (under review) and genetic sequences are available in the European Nucleotide Archive (accession numbers on Appendix 2 in Supplementary Material). Information about study sites (habitat characteristics…) is available through the voucher specimens of which duplicates have been deposited in various herbaria.

## Author Contributions

SI and DBr contributed to conception and design of the study. DBr, TH and LB collected field samples. TH and DBr measured functional traits. TH, DBr, and DBa performed architectural study. LB and AM produced phylogenetical tree. DBr, TH, and LB performed the statistical analysis. DBr and SI wrote the paper. All authors contributed to manuscript revision, read and approved the submitted version.

### Conflict of Interest Statement

The authors declare that the research was conducted in the absence of any commercial or financial relationships that could be construed as a potential conflict of interest.
