# Supplementary material for: Evolution of Plant Architecture, Functional Diversification and Divergent Evolution in the Genus Atractocarpus (Rubiaceae) for New Caledonia
Source: Front Plant Sci. 2018 Dec 4;9:1775. doi: 10.3389/fpls.2018.01775 (PMC6288547; doi:10.3389/fpls.2018.01775)
Supplement: Supplementary file 1 [file Data_Sheet_1.pdf]

Supplementary Material

Evolution of plant architecture, functional diversification and divergent evolution in the genus *Atractocarpus* (Rubiaceae) for New Caledonia

David BRUY*, Tom HATTERMANN, Laure BARRABÉ, Arnaud MOULY, Daniel BARTHELEMY, Sandrine ISNARD

*** Correspondence:** David BRUY: david.bruy@ird.fr

Appendix 1: Mean value and standard deviation of traits values for each sampled species

Taxon	Voucher	Site	GPS point (DD)	Photosynthetic BI	Exploration BI	Fruit volume (cm^3)	SR	Total LA (cm^2)	Mean LA (cm^2)	Leaf number	IN length (cm)	IN diameter (cm)	SLA (cm^2.g^{-1})	SWD (g.cm^{-3})	SSD (g.cm^{-3})	HV	% Pith	% Wood	% Cortex
Atractocarpus sp. nov. 1 Mouly, ined	Bruy 964	Diahoué	164.6894 -20.4851	0,92 ± 0,02	0,81 ± 0,08	53,5 ± 31,3	101 ± 17	18817 ± 4392	154,8 ± 22,3	243,6 ± 125,2	5,1 ± 1,13	0,73 ± 0,03	75,9 ± 4,1	0,71 ± 0,11	0,49 ± 0,04	1475 ± 259	0,24 ± 0,08	0,35 ± 0,1	0,41 ± 0,07
Atractocarpus aragoensis Guillaumin	Bruy 615	Pic Vincent	165.7737 -21.6031	0,29 ± 0,19	0,49 ± 0,15	13,6 ± 6,6	121 ± 32	1477 ± 1175	84,3 ± 29,1	74 ± 39,2	2,37 ± 0,74	0,36 ± 0,04	107,8 ± 6,7	0,65 ± 0,09	0,5 ± 0,07	888 ± 430	0,24 ± 0,05	0,3 ± 0,05	0,46 ± 0,01
Atractocarpus artensis (Montrouz.) Mouly	Bruy 922	Creek bambou	164.3410 -20.4607	0,82 ± 0,04	0,73 ± 0,14	109,6 ± 13,2	110 ± 29	10396 ± 6387	164,1 ± 19,9	219,4 ± 211,7	6,72 ± 2,99	0,68 ± 0,12	98 ± 10,9	0,7 ± 0,09	0,49 ± 0,09	1710 ± 773	0,3 ± 0,07	0,31 ± 0,15	0,39 ± 0,1

Atractocarpus sp. nov. 2 Mouly, ined	Bruy 889	Dogny	165.8778 -21.6217	0,84 ± 0,07	0,67 ± 0,13	11,1 ± 1,6	133 ± 32	3770 ± 2558	53,6 ± 12,9	154 ± 89,5	4,03 ± 1,64	0,45 ± 0,05	91,1 ± 6,6	0,71 ± 0,05	0,5 ± 0,05	501 ± 276	0,28 ± 0,06	0,3 ± 0,07	0,42 ± 0,04
Atractocarpus baladicus (Montrouz. ex Guillaumin & Beauvis.) Mouly	Bruy 650	Tchamba	165.2315 -21.0178	0,01 ± 0,01	0,13 ± 0,07	120,7 ± 61,3	117 ± 17	4619 ± 537	305,6 ± 30,9	21,8 ± 4,9	3,91 ± 1,04	0,81 ± 0,11	69,7 ± 6,8	0,82 ± 0,04	0,5 ± 0,05	4548 ± 517	0,22 ± 0,1	0,41 ± 0,14	0,37 ± 0,05
Atractocarpus bracteatus Schltr. & K.Krause	Bruy 133	Plaine des lacs	166.9035 -22.2751	0,02 ± 0,01	0,11 ± 0,03	218,6 ± 53,2	164 ± 25	4188 ± 1755	144,5 ± 35,5	42,2 ± 8,2	2,46 ± 0,59	0,62 ± 0,04	60,2 ± 2,8	0,77 ± 0,06	0,56 ± 0,05	4104 ± 1748	0,17 ± 0,03	0,37 ± 0,09	0,46 ± 0,07
Atractocarpus brandzeanus (Baill.) Mouly	Bruy 920	Paagoumene	164.1931 -20.4908	0,46 ± 0,19	0,4 ± 0,2	29 ± 9,4	106 ± 25	2299 ± 1116	103 ± 30,5	49 ± 33,5	3,32 ± 1,16	0,47 ± 0,06	83,8 ± 14,4	0,81 ± 0,04	0,52 ± 0,08	1107 ± 312	0,27 ± 0,03	0,26 ± 0,09	0,47 ± 0,08
Atractocarpus sp. nov. 12 Mouly, ined.	Bruy 1042	Nodela	165.3531 -21.4392	NA	NA	NA	NA	NA	NA	NA	NA	NA	NA	NA	NA	NA	NA	NA	NA
Atractocarpus colnettianus (Guillaumin) Mouly	Bruy 154	Roche Ouaième	164.8630 -20.6401	0,02 ± 0,01	0,15 ± 0,09	156,1 ± 109,9	112 ± 39	3791 ± 770	196,2 ± 64,2	32,6 ± 8,4	1,87 ± 0,46	1,09 ± 0,17	41,8 ± 2,7	0,7 ± 0,08	0,43 ± 0,02	3709 ± 739	0,18 ± 0,02	0,3 ± 0,01	0,52 ± 0,03
Atractocarpus confertus (Guillaumin) Mouly	Veillon 5733	Demazure	166.6251 -22.1962	0,01 ± 0,01	0,02 ± 0,01	44,8 ± 8	106 ± 18	16393 ± 5504	922,5 ± 151,5	24,4 ± 6,1	2,46 ± 0,48	1,3 ± 0,12	64,2 ± 5,6	0,7 ± 0,02	0,48 ± 0,03	16314 ± 5514	0,33 ± 0,03	0,28 ± 0,03	0,39 ± 0,03

Atractocarpus sp. nov. 8 Mouly, ined.	Bruy 953	Tchamba	165.2315 -21.0178	0,68 ± 0,06	0,66 ± 0,07	9,4 ± 0,7	117 ± 17	5245 ± 3215	120,4 ± 24,3	99 ± 33,3	3,96 ± 0,62	0,45 ± 0,08	139,9 ± 18	0,77 ± 0,04	0,52 ± 0,05	1505 ± 485	0,3 ± 0,07	0,32 ± 0,06	0,38 ± 0,03
Atractocarpus heterophyllus (Montrouz.) Guillaumin & Beauvis.	Bruy 632	Paagoumene	164.1931 -20.4908	0,12 ± 0,06	0,22 ± 0,09	147,2 ± 36,3	131 ± 30	2349 ± 1368	140,3 ± 47,5	30,2 ± 13,8	2,5 ± 0,63	0,59 ± 0,07	54,1 ± 7,4	0,67 ± 0,07	0,55 ± 0,01	2035 ± 1169	0,21 ± 0,03	0,29 ± 0,08	0,5 ± 0,06
Atractocarpus sp. nov. 13 Mouly, ined.	Barrabé 1513	Barautê	165.2303 -20.9798	0,89 ± 0,03	0,85 ± 0,04	7,4 ± 0,8	114 ± 15	3743 ± 1773	49,9 ± 4,2	181 ± 114,2	4,57 ± 3	0,47 ± 0,04	99,6 ± 15,4	0,75 ± 0,06	0,5 ± 0,06	388 ± 74	0,34 ± 0,07	0,26 ± 0,09	0,41 ± 0,09
Atractocarpus longistipitatus Baill. ex Guillaumin	Bruy 612	Monts Koghis	165.5086 -22.178507	0,0 ± 0,0	0,3 ± 0,12	15,4 ± 3,2	151 ± 39	6417 ± 2650	361,3 ± 110,1	52,2 ± 19,8	1,88 ± 0,7	0,74 ± 0,07	94,3 ± 15,4	0,76 ± 0,04	0,57 ± 0,03	6395 ± 2636	0,15 ± 0,05	0,38 ± 0,08	0,47 ± 0,07
Atractocarpus mollis (Schltr.) Mouly	Bruy 683	Pondandou	165.2173 -20.8171	0,89 ± 0,05	0,74 ± 0,08	8,6 ± 1,1	102 ± 32	8543 ± 5432	86,3 ± 17,3	191,4 ± 48	1,89 ± 0,88	0,4 ± 0,04	159,3 ± 17,3	0,82 ± 0,04	0,45 ± 0,03	787 ± 217	0,25 ± 0,08	0,24 ± 0,05	0,51 ± 0,05
Atractocarpus sp. nov. 6 Mouly, ined.	Bruy 1012	Mont Mou	166.3349 -22.0751	0,6 ± 0,11	0,82 ± 0,06	NA	88 ± 23	5252 ± 2014	190,1 ± 30,3	115,6 ± 39,7	2,64 ± 1,05	0,71 ± 0,05	70,6 ± 9,5	0,72 ± 0,13	0,53 ± 0,07	2174 ± 1137	0,26 ± 0,06	0,25 ± 0,12	0,49 ± 0,08
Atractocarpus ngoyensis (Schltr.) Mouly	Bruy 139	Plaine des lacs	166.9035 -22.2751	0,4 ± 0,13	0,49 ± 0,16	169,4 ± 39,1	140 ± 13	6580 ± 2245	228,9 ± 36,9	85 ± 47	3,64 ± 1,63	0,77 ± 0,1	70,7 ± 7,8	0,81 ± 0,04	0,58 ± 0,16	3740 ± 758	0,2 ± 0,06	0,38 ± 0,06	0,42 ± 0,04

Atractocarpus nigricans (Schltr.) Mouly	Bruy 662	Roche Ouaième	164.8630 -20.6401	0,86 ± 0,07	0,66 ± 0,16	17,3 ± 4,3	115 ± 13	6714 ± 3161	135,3 ± 31,8	79,4 ± 42,6	4,79 ± 3,41	0,63 ± 0,05	92,4 ± 5	0,73 ± 0,11	0,43 ± 0,04	792 ± 190	0,29 ± 0,06	0,2 ± 0,07	0,51 ± 0,04
Atractocarpus sp. nov. 4 Mouly, ined.	Bruy 929	Mandjelia	164.5421 -20.4038	0,69 ± 0,11	0,68 ± 0,06	3,9 ± 0,6	144 ± 37	1294 ± 200	36,9 ± 7,5	85,2 ± 33	2,39 ± 1,56	0,35 ± 0,01	82,6 ± 3,7	0,79 ± 0,05	0,51 ± 0,04	409 ± 156	0,25 ± 0,07	0,26 ± 0,06	0,5 ± 0,03
Atractocarpus sp. nov. 10 Mouly, ined.	Bruy 944	Pwaala	164.5087 -20.3750	0,92 ± 0,04	0,85 ± 0,02	11,2 ± 6,6	101 ± 16	10925 ± 3546	95,5 ± 34	148 ± 60,4	1,96 ± 0,61	0,45 ± 0,05	157,4 ± 7,6	0,79 ± 0,02	0,53 ± 0,03	833 ± 411	0,26 ± 0,03	0,3 ± 0,03	0,44 ± 0,04
Atractocarpus sp. nov. 3 Mouly ined.	Barrabé 699	Aoupinié	165.2769 -21.1780	0,87 ± 0,07	0,84 ± 0,06	10,5 ± 3,3	122 ± 22	9511 ± 3455	142,2 ± 44,1	140 ± 62,8	5,25 ± 2,39	0,7 ± 0,1	172,1 ± 36,6	0,68 ± 0,09	0,42 ± 0,05	1126 ± 585	0,34 ± 0,08	0,27 ± 0,12	0,39 ± 0,06
Atractocarpus pseudoterminalis (Guillaumin) Mouly	Bruy 162	Plaine des lacs	166.9035 -22.2751	0,78 ± 0,12	0,76 ± 0,14	13,5 ± 4,1	160 ± 13	2933 ± 1461	44,4 ± 7,6	186,2 ± 138,1	3,46 ± 1,28	0,32 ± 0,01	88,9 ± 5,5	0,81 ± 0,03	0,58 ± 0,04	517 ± 49	0,21 ± 0,07	0,37 ± 0,11	0,42 ± 0,06
Atractocarpus pterocarpon (Guillaumin) Puttock	McPherson 3003	Plaine des lacs	166.9035 -22.2751	0,01 ± 0,01	0,26 ± 0,11	30,4 ± 11,6	127 ± 28	2321 ± 701	189 ± 22,5	38,8 ± 21,6	2,57 ± 1,02	0,6 ± 0,05	76,3 ± 4,3	0,75 ± 0,06	0,5 ± 0,02	2304 ± 698	0,19 ± 0,03	0,25 ± 0,05	0,55 ± 0,02
Atractocarpus sp. nov. 7 Mouly, ined.	Hattermann 01	Mont Do	166.0008 -21.7536	0,48 ± 0,08	0,4 ± 0,11	33,8 ± 17,2	177 ± 31	1610 ± 581	63,5 ± 10	46 ± 18,5	3,37 ± 1,77	0,58 ± 0,12	63,2 ± 12,2	0,72 ± 0,08	0,48 ± 0,05	842 ± 351	0,22 ± 0,05	0,33 ± 0,09	0,45 ± 0,07

Atractocarpus sessilifolius Guillaumin	Bruy 645	Tchamba	165.2315 -21.0178	0,15 ± 0,14	0,53 ± 0,13	6,5 ± 5,6	98 ± 16	1872 ± 738	113,7 ± 36	80,4 ± 25,7	1,99 ± 1,3	0,45 ± 0,08	67,4 ± 10,8	0,8 ± 0,03	0,59 ± 0,03	1641 ± 828	0,1 ± 0,06	0,37 ± 0,04	0,53 ± 0,06
Atractocarpus sezitat (Guillaumin) Mouly	Bruy 900	Roche d'Adio	165.2422 -21.2405	NA	NA	NA	NA	NA	NA	NA	NA	NA	NA	NA	NA	NA	NA	NA	NA
Atractocarpus vaginatus Guillaumin	Bruy 621	Sarramea	165.8072 -21.6658	0,09 ± 0,1	0,07 ± 0,03	360,7 ± 110,9	128 ± 16	3754 ± 1003	229,7 ± 26,8	24 ± 7,3	6,58 ± 1,13	0,82 ± 0,08	40,7 ± 5,3	0,82 ± 0,15	0,59 ± 0,23	3356 ± 718	0,19 ± 0,04	0,35 ± 0,04	0,46 ± 0,03

BI: branching index, SR: slenderness ratio, LA: leaf area, IN: internode, SLA: specific leaf area, SWD: specific wood density, SSD: specific stem density, HV: Huber value

Appendix 2: Accession numbers for DNA sequence data used in the study

Species name	Herbarium voucher (Institution acronym)	ITS (accession number)	rpl32 (accession number)	trnTF (accession number)
Atractocarpus aragoensis Guillaumin	Munzinger 3539 (NOU)	MK165281	MK123862	MK165233
Atractocarpus artensis (Montrouz.) Mouly, ined.	Barrabé 755 (NOU)	MK165309	MK123883	MK165260
Atractocarpus baladicus (Guillaumin) Mouly, ined.	Mouly 813 (P)	MK165310	MK123884	MK165261
Atractocarpus brandzeanus (Baill.) Mouly	Barrabé 352 (NOU)	MK165311	MK123885	MK165262
Atractocarpus bracteatus Schltr. & K.Krause	Mouly 213 (P)	MK165282	MK123863	MK165234
Atractocarpus colnettianus (Guillaumin) Mouly, ined.	Bruy 154 (NOU)	MK165283	MK123864	MK165235
Atractocarpus confertus (Guillaumin) Mouly, ined.	Barrabé 330 (NOU)	MK165305	MK123880	MK165257
Atractocarpus fitzalanii (F.Muell.) Puttock	Puttock 88000767 (S)	MK165285	NA	MK165237
Atractocarpus heterophyllus (Montrouz.) Guillaumin & Beauvis.	Mouly 850 (P)	MK165286	KJ136904	MK165238
Atractocarpus hirtus (F.Muell.) Puttock	Forsberg 61489 (P)	MK165287	NA	MK165239
Atractocarpus longistipitatus Baill. ex Guillaumin	Mouly 742 (P)	MK165288	MK123865	MK165240
Atractocarpus merikin (F.M.Bailey) Puttock	Webb 11457 (P)	MK165289	NA	MK165241
Atractrocarpus mollis (Schltr.) Mouly, ined.	Mouly 808 (P)	MK165307	KJ816154	MK165259
Atractocarpus ngoyensis (Schltr.) Mouly, ined.	Munzinger 1565 (P)	MK165313	MK123887	MK165264
Atractocarpus nigricans (Schltr.) Mouly, ined.	Mouly 23 (P)	MK165314	MK123888	MK165265
Atractocarpus pseudoterminalis (Guillaumin) Mouly, ined.	Munzinger 2256 (NOU)	MK165316	MK123890	MK165267
Atractocarpus pterocarpon (Guillaumin) Puttock	Munzinger 2314 (NOU)	MK165308	MK123882	KF965231
Atractocarpus sessilifolius Guillaumin	Mouly 330 (P)	MK165291	MK123867	MK165243
Atractocarpus sezitat (Guillaumin) Mouly, ined.	Barrabé 716 (NOU)	MK165317	MK123891	MK165268
Atractocarpus sp1	Munzinger 1631 (NOU)	MK165292	MK123868	MK165244
Atractocarpus sp2	Bruy 889 (NOU)	MK165315	MK123889	MK165266
Atractocarpus sp3	Mouly 810 (P)	MK165293	MK123869	MK165245
Atractocarpus sp4	Bruy 311 (NOU)	MK165294	MK123870	MK165246
Atractocarpus sp6	Barrabé 743 (NOU)	MK165295	MK123871	MK165247
Atractocarpus sp7	Barrabé 661 (NOU)	MK165296	MK123872	MK165248
Atractocarpus sp8	Bruy 953 (NOU)	MK165297	MK123873	MK165249
Atractocarpus sp10	Bruy 944 (NOU)	MK165299	MK123875	MK165251

Atractocarpus sp12	Bruy 1042 (NOU)	MK165301	MK123877	MK165253
Atractocarpus sp13	Barrabé 1513 (NOU)	MK165302	MK123878	MK165254
Atractocarpus vaginatus Baill. ex Guillaumin	Mouly 831 (P)	MK165304	MK123879	MK165256
Bungarimba ridsdalei K.M.Wong	Wong 2875 (KLU)	MK165323	KJ816128	KF965170
Catunaregam spinosa (Thunb.) Tirveng.	Luke 8332A (S)	MK165325	MK123894	KF965178
Porterandia anisophylla (Jack ex Roxb.) Ridl.	Zahid 28 (KLU)	MK165335	MK123901	KF965243
Randia moorei F.Muell. ex Benth.	Leiper s.n. (BRI)	MK165312	MK123886	MK165263
Rubovietnamia aristata Tirveng.	Bastien VN352 (P)	MK165338	MK123903	KF965254
Sukunia pentagonioides Seem.	Smith 8376 (S)	MK165339	KJ816130	KF965266
Trukia carolinensis Valeton	Fosberg 60242 (P)	MK165342	MK123905	KF965274
Vidalasia fusca (Craib) Tirveng.	Larsen 43277 (P)	MK165344	KJ816132	KF965275

NA: sequence non available
